# Basic cardiopulmonary resuscitation knowledge of house-officers in a tertiary institution: factors determining accuracy

**DOI:** 10.11604/pamj.2014.18.209.3654

**Published:** 2014-07-08

**Authors:** Kelechi Emmanuel Okonta, Boma Alali Ngozi Okoh

**Affiliations:** 1 Department of Surgery, University of Port Harcourt Teaching Hospital, Rivers State, Nigeria; 2 Department of Paediatrics and Child Health, University of Port Harcourt Teaching Hospital, Rivers State, Nigeria

**Keywords:** Resuscitation, house-Officers, training, tertiary Institution, Port-Harcourt

## Abstract

**Background:**

To assess the level of knowledge of CPR among House-Officers (HO) and some factors determining accuracy of knowledge.

**Methods:**

A total of 50 structured questionnaires were administered to HO with 35 (70%) questionnaires duly filled and returned. Data on the participants’ brief biodata and the understanding of basic skills of BLS were collected and analyzed using International Business Machine SPSS Statistics version 21 for Windows. The t-test for independent samples was applied for the grouped data with P < 0.05 taken as level of significance.

**Results:**

The age of the respondents ranged from 20-37 years with the mean age of 25.4 + SD2.7 years and the male/female ratio was 1:1.3. Eleven (31.4%) out of the 35 HO had no prior CPR training while 68.6% had prior training; Eighteen (51.4%) had training within the last 2 years. Twenty (57.1%) had performed CPR in a real situation, while 42.9% had not. Six (17.1%) HO scored above 50% while 82.9% scored below 50%. The female HO got more correct answers than the males (25% versus 6.7%, p = 0.167). The number of respondents who had prior CPR training had more correct answers than those who did not (25% versus 0%, p = 0.083) while those who had previously performed CPR had more correct answers than those who had not (33.3% versus 5%, P <0.05).

**Conclusion:**

There was a general poor knowledge of the performance of basic CPR amongst HOs. However, previous experience of having performed CPR in real setting, or the use of mannequins, improved their theoretical knowledge of CPR.

## Introduction

Internship is an important land mark in any doctor's career, as it enables the doctor work in a wide range of departments and sees patients in different specialties during rotations through the different departments [1]. In teaching hospitals, the responsibilities of performing Cardiopulmonary resuscitation (CPR) rest largely on the House-Officers (HOs), yet most of them do not receive formal training on the conduct of CPR [2, 3]. Thus, teaching Basic Life Support (BLS) skills in the teaching hospitals should be taken seriously. In fact, the teaching should be targeted at the groups most likely to actually apply the acquired skill in order to make best use of the resources available [4] and HOs are one of the important groups suited for this task [5]. Also, these HOs should be followed up with regular training in CPR as well as the practice of CPR on mannequins before they have primary responsibility of CPR [2] The knowledge, and being able to administer CPR properly, when required, is a necessary perquisite for the HO and indeed, even before the commencement of internship; as the success of resuscitation of patients depends on timely and proper mode of administration of CPR. From previous studies, it was observed that the probability of survival was greater when BLS was begun within 4 minutes of arrest than when it was begun after 4 minutes; regardless of whether advanced cardiac life support was begun within 10 minutes [6]. The putative findings from previous studies are that the understanding and ability to perform CPR amongst HOs are poor in both BLS and advanced Life Support( ALS) [3, 7]. And the reasons for this poor knowledge of CPR stems from no prior training on the performance to the stressful nature of the conduct of CPR [2–8]. In one study however, a slight exception was observed among HOs in internal medicine and that was because of their completion of life support courses which increased their overall knowledge and their confidence of administering CPR [8]. The aim of this study is to assess the level of knowledge of CPR among House Officers in a tertiary institution, and some factors determining accuracy of knowledge.

## Methods

The study was a cross sectional survey of House officers in the University of Port Harcourt Teaching Hospital, Nigeria (the authors’ institution). Questionnaires were distributed among all the House officers of the Hospital, who had given verbal consent, indicating interest to be part of the study and house officers who failed to return their questionnaires or incompletely filled questionnaires were excluded. The questionnaire was self administered, anonymous, and prepared in English language. It inquired about basic biodata of the subjects and information on previous CPR training and performance. It also included thirteen practical test questions on CPR knowledge and skill. The practical questions covered areas on BLS abbreviation, recommended order of CPR technique, location, rate and depth of chest compression, how to give rescue breaths and coordinate with chest compressions and removal of airway foreign body obstruction. The purpose of the study was explained prior to filling the questionnaires A structured questionnaire was administered to collect data on the participants’ biodata and the understanding of basic skills of BLS. The mean and standard deviations (SD) for the age and the data from the responses were analyzed using International Business Machine SPSS Statistics version 21 for Windows. The t-test for independent samples was applied for the grouped data with P < 0.05 taken as level of significance.

## Results

A total of 50 structured questionnaires were administered to House Officers who were at the moment doing their postings in the different departments with 35 (70%) questionnaires duly filled and returned. The basic knowledge on the conduct of CPR was evaluated for 15 minutes as provided by the questionnaires administered. The age of the respondents ranged from 20 to 37 years with the mean age of 25.4 + SD2.7 years and the male to female ratio of 1:1.3 (Table 1). Eleven (31.4%) out of the 35 house officers had no prior CPR training while 68.6% had prior training ((53.6% in auxiliary medical service, 10.5 in a teaching hospital and 4.5% from other sources); 51.7% had training within the last 2years). Twenty (57.1%) of the 35 house officers had performed CPR in a real situation, while 42.9% had not. The summary of the answers showed that only 6 (17.1%) of the 35 respondents scored above 50% while 82.9% scored below 50%. Using the sex, the female respondents got more correct answers than their male counterparts (25% versus 6.7%, p =0.167). The number of respondents who had prior CPR training had more correct answers than those who did not (25% versus 0%, p =0.083) while those who had previously performed CPR had more correct answers than those who had not (33.3% versus 5%, P <0.05) (Table 2).

**Table 1:** Age group and sex distribution of respondents

	Male	Female	Total
**<20**	0 (0.0)	0 (0.0)	0 (0.0)
**20-24**	2 (15.4)	11 (84.6)	13 (37.1)
**25-29**	12 (57.1)	9 (42.9)	21 (60.0)
**>29**	1 (100.0)	0 (0.0)	1 (2.9)
**Total**	15	20	35

**Table 2:** Relationship between respondents’ scores and some parameters

Parameter		Score		χ^2^	p value
		>50%	<50%		
		No (%)	No (%)		
**Sex**	Male	1 (6.7)	14 (93.3)	2.03	0.167
	Female	5 (25.0)	15 (75.0)		
**Prior CPR training**	Yes	6 (25.0)	18 (75.0)	3.32	0.083
	No	0 (0.0)	11 (100.0)		
**Prior CPR performance**	Yes	5 (33.30)	10 (66.7)	4.84	0.04
	No	1 (5.0)	19 (95.0)		

All(100%) the HO gave the correct answer as to the abbreviation of BLS; 22 respondent (62.9%) did not know the current order of CPR;23(65.7%) did not know what to do if a patient was unresponsive at first;33( 94.3%) did not know what to do if patient still remained unresponsive even after shaking and shouting;14(40%) were able to locate the area of chest compression in adults;13(37.4%) were able to locate the area of chest compression in infants;26(74.3%) did not know what to do if they did not want to give mouth-to-mouth breath;9( 25.7%) knew how to give rescue breathing in infants;12( 34.3%) knew the depth of chest compression in adults;16(45.7%) knew the rate of chest compression in adults and children; 9(25.7%) knew the ratio of CPR in single rescuer of an adult; 16(45.7%) knew the chest compression and ventilation ratio in a new born; 27(77.1%) knew when to perform Heimlich manoeuvre.

## Discussion

Overall, the HOs performed poorly and showed grossly insufficient knowledge on basic CPR. This was the finding of Jensen et al [7] who showed that newly graduated doctors do not have sufficient competence in managing cardiopulmonary arrest. Similarly, other studies showed poor knowledge of HOs in performance of CPR [9, 10]. However, all the HOs gave the correct answers to the meaning of the abbreviation of BLS (Basic Life Support); the implication is that the HOs were aware of this procedure or at least know that such appears in the armamentaria of patients’ care guidelines. Yet quite a large number did not know the current order of CPR. Though no previous study had deliberately compared the effect of sex on the performance of CPR, however from our review, the female HOs gave more correct answers to the correct performing of CPR. This is in apparent contrast to a work done in Norway which showed that female house officers were less likely than their male colleagues to demonstrate these various practical clinical skills [11]. On the stressful effect of CPR, Morgan et al suggested that most junior doctors feel stressed from CPR performance, and adequate training, improving communication skills, and support for junior doctors in the cardiac arrest team therefore, needs to be reviewed, since improvement in these areas may help to reduce stress [3].

One-fourth of those who had prior training on CPR passed the questions compared to none, in those who had not had prior CPR training. Those who had previous training on CPR and having performed CPR had higher correct answers than those who had not had either. This was the finding from a previous study that showed that HO who received prior training tend to perform better in BLS (p < 0.001) [2]. In another survey using paediatric residents’ knowledge on resuscitation, Hunt et al [12] by applying a multivariate analysis showed that the level of training, amongst others were not independently associated with resuscitating appropriately, whereas attendance at an institutional Code Team training course was. Also, work done by Donogrio et al^8^ showed that a previous formal training in cardiac life support was found to be associated with a higher level of confidence in the ability to administer cardiac lifesaving techniques (p <0.0001) and a higher overall knowledge score (p = 0.003). Furthermore, Seilden et al [10] recommended that training of the HO should be in conjunction with other members of the arrest team as this may lead to improved survival of patients after cardiac arrest. About less than 57.1% had received training within the last 2 years, Goodwin et al [9] recommended that junior hospital doctors should undergo regular CPR training every 6 months, in order to maintain their practical CPR skills.

The impact of performing CPR previously on patients on the knowledge of CPR was demonstrated in our study. There were a statistically significant percentage of 33.3% respondents from those who had actually performed CPR on patients scoring above average as compared to 5% of those who had not previously performed CPR. It is known that cardiopulmonary arrests tend to generate anxiety among HO, so with additional teaching and practice in actual performance of CPR, the comfort levels among HO will increase when they are given the opportunity to practice these skills [13]. Prior training will lead to acquisition of the skill and furthermore lead to confidence in the performance of CPR and performing it with reduced anxiety on patients [3, 8].

### Limitations

The departments of the HOs at the time of the rotation/posting were not requested nor the posting previously completed. These would have possibly allowed the assessment of the impact of the various departments on their ability and knowledge of CPR. Practical simulations test would have been apt in really evaluating of their understanding of CPR.

## Conclusion

From the study, there is a general poor knowledge of the performance of basic CPR amongst HOs. However, previous experience of having performed CPR in real setting, or the use of mannequins, improved their theoretical knowledge of CPR. Thus the responsibility of a tertiary institution should be the organization of training sessions for HO and ensuring the performance of CPR before the commencement of internship.
